# Reciprocity in spatial evolutionary public goods game on double-layered network

**DOI:** 10.1038/srep31299

**Published:** 2016-08-09

**Authors:** Jinho Kim, Soon-Hyung Yook, Yup Kim

**Affiliations:** 1 Department of Social Network Science, Kyung Hee University, Seoul 130-701, Korea; 2 Department of Physics and Research Institute for Basic Sciences, Kyung Hee University, Seoul 130-701, Korea

## Abstract

Spatial evolutionary games have mainly been studied on a single, isolated network. However, in real world systems, many interaction topologies are not isolated but many different types of networks are inter-connected to each other. In this study, we investigate the spatial evolutionary public goods game (SEPGG) on double-layered random networks (DRN). Based on the mean-field type arguments and numerical simulations, we find that SEPGG on DRN shows very rich interesting phenomena, especially, depending on the size of each layer, intra-connectivity, and inter-connected couplings, the network reciprocity of SEPGG on DRN can be drastically enhanced through the inter-connected coupling. Furthermore, SEPGG on DRN can provide a more general framework which includes the evolutionary dynamics on multiplex networks and inter-connected networks at the same time.

Cooperation is a ubiquitous phenomenon in nature from micro-organisms to human society. The emergence of cooperation among the selfish individuals has been a long lasting conundrum in various scientific disciplines^1^,^2^,^3^,^4^,^5^,^6^,^7^,^8^,^9^,^10^,^11^,^12^. There have been many attempts to explain how the cooperation emerges through the interaction among selfish individuals. Among those studies game theory has provided an important theoretical framework to understand the emergence of cooperation through the strategic interactions among individuals. And it has been successfully applied to diverse fields such as evolutionary biology and psychology^1^, computer science and operations research^2^,^3^, political science and military strategy^4^,^5^, cultural anthropology^6^, ethics and moral philosophy^7^, economics^8^,^9^, traffic flow^10^,^11^ and public health^12^. The central aim of game theory is to determine conditions needed for cooperation to emerge between egoistic individuals^13^,^14^,^15^. Recently, many studies have focused on spatial evolutionary games to understand how steady-state strategies emerge in various structures and to identify the characteristic features of steady-state strategies^15^,^16^,^17^,^18^. Interestingly, in structured population, individuals only interact with their nearest neighbors and it becomes possible for cooperators to survive by forming clusters in which they defend themselves against defectors’ exploitation. This is known as network reciprocity^16^,^17^,^18^,^19^,^20^,^21^,^22^,^23^,^24^,^25^.

Spatial evolutionary games have mainly been studied on a single, isolated network. However, empirical evidences show that many, if not all, real world systems are not isolated but many different types of networks are interlinked^26^. For example, family, friendship and work-related networks are interlinked by each individual in society. Metabolic synthesis, protein-protein interaction, signaling and regulatory networks altogether constitute an inter-cellular network in a cell. Various financial, trade and political networks are also interlinked to form a global economic system. The ecological system is also composed of different level of hierarchical networks. Thus, the game theory on a single network cannot provide a complete explanation on how the cooperation emerges in nature. Only recently, spatial evolutionary games on the interdependent networks, multiplex networks, and interconnected networks have been studied to understand how imitation and interaction between the networks influence the final cooperation levels^26^,^27^,^28^,^29^,^30^,^31^,^32^.

In the interconnected network, there are actual physical links between different networks rather than the dependency links in interdependent networks. Propagation of microcredit across the countries, possibly across the interconnected networks is an example of the propagation of cooperations from one network to another. In Bangladesh, microcredit has grown in popularity in the 1970s. Group-lending is a key part of microcredit. The loan to one participant in group-lending depends upon the successful repayment from another member, thus cooperation among participants is very important in microcredit. After few years, microcredit is widely used in developing countries and is presented as having “enormous potential as a tool for poverty alleviation”^33^. In 2007, there are more than 500 organizations in the United States that provide to microcredit owners^34^.

To understand how the coupling between networks develops the cooperation or the network reciprocity, in this report, we investigate the spatial evolutionary public goods game (SEPGG) on double-layered random networks (DRN’s). Especially, the spatial evolutionary public goods game (SEPGG) has attracted considerable attention, because it offers valuable insights into prevailing socioeconomic problems such as pollution, deforestation, mining, fishing, climate control and environmental protection^18^. SEPGG has been intensively studied to find interesting results such as the cyclic dominance^35^,^36^, transition nature^37^, payoff distribution^38^, and the effects of underlying topologies^19^,^26^,^27^,^28^,^29^,^30^,^31^,^38^,^39^,^40^,^41^,^42^,^43^,^44^. The effects of loner^39^,^45^, punishment and reputation^46^, and noise^47^ on the emergence of cooperation have been also investigated. As a prototype of interconnected network, we consider a double-layered random networks (DRNs), but the generalization to interconnected network with more than two layers is straightforward. A DRN consists of two random networks with any size and average degree. If the size of each layer is the same then the DRN can be regarded as a multiplex network, in which each layer has the same set of nodes. On the other hand, if the size of each layer is different, then each node of one random network with the smaller size is linked to a randomly chosen node of the other network. In this case the DRN can be regarded as an interconnected network in which two different networks are interlinked through the interconnected links. Therefore, the DRN considered in this study can provide a more general framework to investigate the emergence of cooperation in various types of interlinked networks. We use the biased imitation process^24^, in which a randomly chosen agent imitates the strategy of the interlinked neighbor on the opposite layer with probability *p*, or that of a randomly chosen node among intralinked neighbors on the same layer with the probability 1 − *p*. By simulations, we obtain the steady-states depending on *p*. Especially, for *p* > 0, we find that the anomalous cooperator-enhanced states on the layer which have no cooperators for *p* = 0. This anomalous cooperator-enhance states resembles the propagation of microcredit. We also explain theoretically how this network reciprocity occurs.

## Previous Study

To understand “Tragedy of the commons”^48^ problem with large participants has been studied through the SEPGG on the complete graph (CG) and dense random networks^17^. Depending on the multiplication factor *r* and the size of graph *N*, either **Loner-only state (L-state)**, which is the anomalous state with no active participants, or **Defector-only state (D-state)**, which means the state of “tragedy of the commons”, has been shown to appear on CG^17^. Furthermore, we have shown the following crossover behaviors as the mean-degree 〈*k*〉 of underlying random networks changes^17^. For small *r*, the L-state crosses over to the D-state and the D-state successively crosses over to the **Cooperator-only state (C-state)** as 〈*k*〉 decreases. For large *r*, the direct crossover from the D-state to the C-state occurs as 〈*k*〉 decreases. We have been found that cooperation gradually increases as the number of participants or 〈*k*〉 decreases, which is the origin of these crossovers. Hence, the crossovers describe how the enhanced cooperation on sparse networks overcomes “tragedy of the commons” on dense networks.

## Results

### SEPGG on double-layered random network

**Figure 1:**
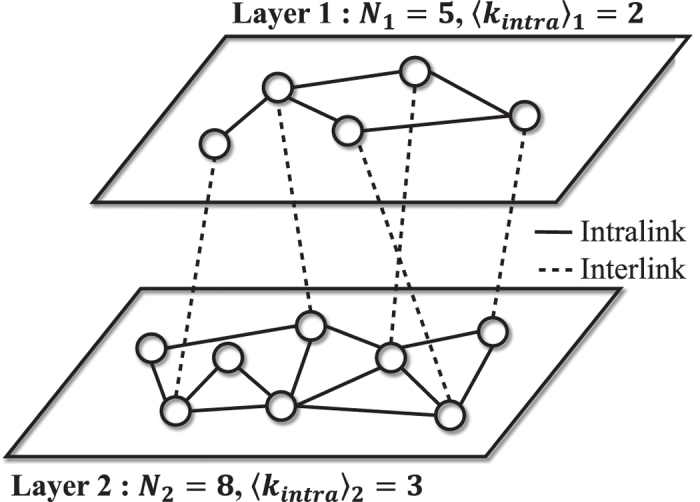
Schematic diagram for the construction of a DRN. The first random layer with the size *N*_1_ and the average intradegree 〈*k*_*intra*_〉_1_ and the second random layer with *N*_2_(≥*N*_1_) and 〈*k*_*intra*_〉_2_ are separately constructed. To construct random layers we use the Erdös-Rényi (ER) network model^57^ whose degree distribution is known to satisfy the Poisson distribution. Then, for DRN with *n*_*inter*_(≤*N*_1_) interlinks, *n*_*inter*_(≤*N*_1_) different nodes are randomly chosen on both layers. Then, *n*_*inter*_ links are made, so that one-to-one correspondence between the chosen nodes on the first layer and those on the second layer occurs.

Now we want to explain how the **double-layered random networks (DRNs)** are composed. The first random network (layer) with the size *N*_1_ and the average intradegree 〈*k*_*intra*_〉_1_ and the second random layer with *N*_2_(≥*N*_1_) and 〈*k*_*intra*_〉_2_ are separately constructed. Then, to make DRN with *n*_*inter*_(≤*N*_1_) interlinks, *n*_*inter*_ different nodes both on the first layer and the second layer are chosen randomly. Each chosen node on the layer 1 is made to be randomly linked to a chosen node of the second layer without making multiple interlinks to a certain node. (See Fig. 1). We call a DRN with *N*_1_ = *N*_2_(=*N*) and 〈*k*_*intra*_〉_1_ = 〈*k*_*intra*_〉_2_ a **symmetric DRN** and a DRN with *N*_1_ ≤ *N*_2_ or 〈*k*_*intra*_〉_1_ ≠ 〈*k*_*intra*_〉_2_ an **asymmetric DRN**.

SEPGG model on a constructed DRN is defined as what follows. Each agent is assigned to a node on DRN. The strategy *s*_*i*_ of the agent on a node *i* can be Cooperator (C), Defector (D) or Loner (L). In each update an agent *i* is randomly chosen. First, we calculate the payoff *P*_*i*_ of *i* using the following rule. Let *n*_*i*,*C*_ be the number of agents with C, *n*_*i*,*D*_ be that with D and *n*_*i*,*L*_ be that with L among the *k*_*i*,*intra*_ + *k*_*i*,*inter*_ + 1 agents. Here *k*_*i*,*intera*_ (*k*_*i*,*inter*_) is the intradegree (interdegree) of node *i* and *n*_*i*,*C*_ + *n*_*i*,*D*_ + *n*_*i*,*L*_ = *k*_*i*,*intra*_ + *k*_*i*,*inter*_ + 1. *P*_*i*_ is given by,


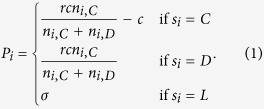


Here, *c* is the cost contributed to the common pool by a C, *r*(>1) is the multiplication factor and *σ* is the fixed payoff of an L. We imposed the condition 0 < *σ* < *c*(*r* − 1)^35^. Then, *i* changes its strategy through a biased imitation process as what follows^24^. With the probability *p*, the interlinked neighbor *j* on the opposite layer is selected. With the probability 1 − *p*, an intralinked neighbor *j* on the same layer is randomly selected. If *i* has no interlink, we choose a neighbor *j* from the same layer regardless of *p*. The strategy of *i* is changed into the strategy of *j* with the transition probability *f*_*ij*_, where


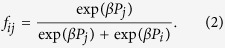


Here, *β*(≥0) controls the amount of noise. When *β* → 0 *i* randomly adopts the strategy of *j*. However, for *β* > 0 we have shown that there exist distinctive states on random network^17^. As summarized below the steady-state depends only on 
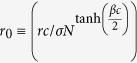
 and 〈*k*_*intra*_〉^17^. Therefore, in this paper, we mainly use *β* = *c* = *σ* = 1 for the numerical analyses without loss of generality.

### Results on the DRN with *N*_1_ = *N*_2_ = *N* and *k*_*inter*_ = 1

Let’s first study the SEPGG model on the DRN with *N*_1_ = *N*_2_ = *N* on which any agent *i* has one interlink or *k*_*i*,*inter*_ = 1. We focus the steady-state densities 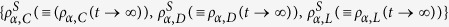 under the initial condition 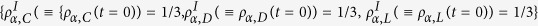. Here, *ρ*_*α*,*C*_(*t*) means the density of C on the layer *α*(=1, 2) at the time *t* and etc. All quantities are averaged over 2000 realization of networks. For each network realization strategies are randomly assigned to each agent with given 
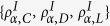
.

When *p* = 0, there exists no coupling between the layers and the steady-states of each layers are the same as those on a single random network, which we have already studied in ref. 17. The followings are brief summary of previous results. Depending on *r* and 〈*k*〉_*α*_(=〈*k*_*intra*_〉_*α*_ + 〈*k*_*inter*_〉_*α*_), the steady-state on each layer for *p* = 0 becomes one of the following 5 states. For *r*_0_ = 0.3(<1)

- **C-state with** 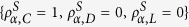 for 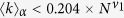 with 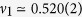.
- **D-state with** 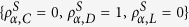 for 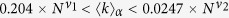 with 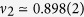.
- **L-state with** 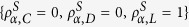 for 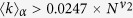.For *β* = *c* = *σ* = 1 and 
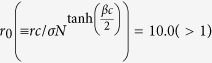
- ** C-state with** 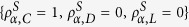 for 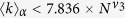 with 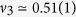.
- **D-state with** 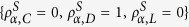 for 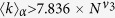.

Next we consider the case of *p* = 1(complete coupling). In complete coupling, any pair of interlinked nodes with identical strategies, i.e., a C-C pair, a D-D pair or an L-L pair, cannot be changed. Any pair with different strategies should change into a pair with identical strategies by the first successful transition. Therefore, the final steady-state is the absorbing state in which any interlinked pair has a common strategy and 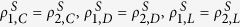. Considering the final absorbing state under the initial condition, 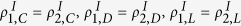, there cannot be anomalous effects to break the density symmetry between layers during evolution. So, we expect *ρ*_1,*C*_(*t*) = *ρ*_2,*C*_(*t*), *ρ*_1,*D*_(*t*) = *ρ*_2,*D*_(*t*), *ρ*_1,*L*_(*t*) = *ρ*_2,*L*_(*t*) for *p* = 1, which is confirmed by simulations on various DRNs. To understand the steady-state behavior for *p* = 1 in a mean-field level, we study the PGG model on the symmetric DRN of two complete graphs (CGs). The payoff of a node on CG simply depends on *ρ*_*C*_, *ρ*_*D*_ and *ρ*_*L*_^17^. When 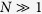, the payoff of a node with D on both CG 1 and 2 is written as 
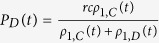
 and the payoff of a node with *C* is *P*_*C*_(*t*) = *P*_*D*_(*t*) − *c*. Therefore, the transition probability *f*_(1,*D*)(2,*C*)_(*t*) that a D-node on the CG 1 accepts the C-strategy of the interlinked node on the CG 2 is equal to *f*_(2,*D*)(1,*C*)_ and 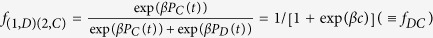. Likewise *f*_*CD*_(≡*f*_(1,*C*)(2,*D*)_ = *f*_(2,*C*)(1,*D*)_) = exp(*βc*)/[1 + exp(*βc*)], *f*_*LD*_(≡*f*_(1,*L*)(2,*D*)_ = *f*_(2,*L*)(1,*D*)_) = 1/[1 + exp(*β*(*σ* − [*rcρ*_1,*C*_(*t*)]/[*ρ*_1,*C*_(*t*) + *ρ*_1,*D*_(*t*)])) and *f*_*LC*_(≡*f*_(1,*L*)(2,*C*)_ = *f*_(2,*L*)(1,*C*)_) = 1/[1 + exp(*β*(*σ* − [*rcρ*_1,*C*_(*t*)]/[*ρ*_1,*C*_(*t*) + *ρ*_1,*D*_(*t*)] + *c*))]. Similarly, *f*_*DL*_(≡*f*_(1,*D*)(2,*L*)_ = *f*_(1,*L*)(2,*D*)_ = 1 − *f*_*LD*_) and *f*_*CL*_(≡*f*_(1,*C*)(2,*L*)_ = *f*_(1,*L*)(2,*C*)_ = 1 − *f*_*LC*_) are obtained. Since the first successful update changes an interlinked pair of nodes with different strategies (**active pair**) into the pair with the same strategies (**dead pair**), the final absorbing state appears very rapidly. If the active pair is changed into the dead pair in accordance with the initial transition probabilities, then the steady-state for *p* = 1 on the DRN of two CGs is calculated by


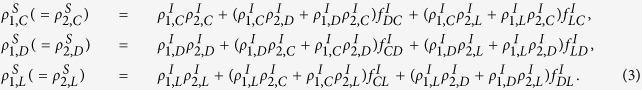


**Figure 2:**
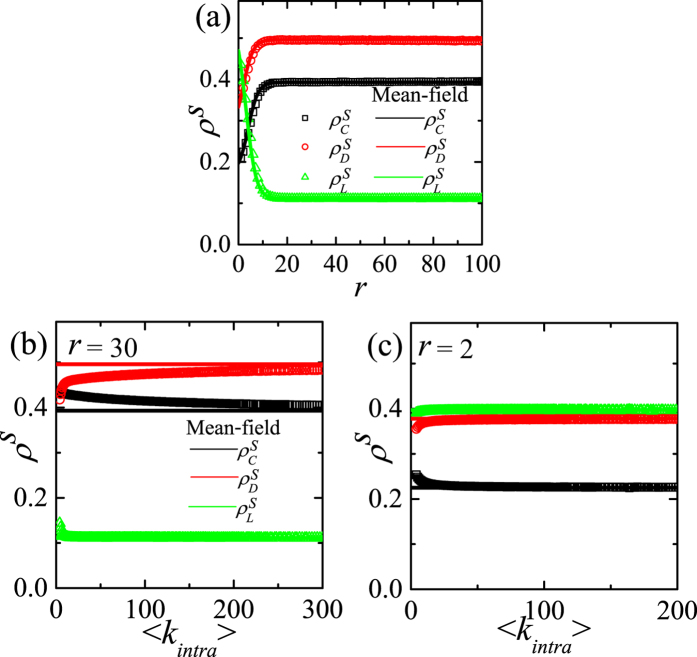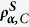, 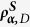 and 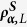 on the symmetric DRNs with *N* = 16,000 for *p* = 1. (**a**) Plots of 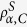, 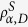 and 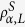 on the DRN of two complete graphs against *r*. The lines represent the mean-field results from equation (3). (**b**) Plots of 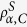, 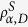 and 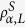 against 〈*k*_*intra*_〉_1_(=〈*k*_*intra*_〉_2_) of the symmetric DRN for *r* = 30.0. The horizontal lines represent the mean-field results. (**c**) Plots of 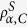, 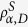 and 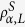 and mean-field results for *r* = 2.0. The symbols and lines standing for *ρ*^*S*^’s in this figure are used commonly in any plot in this paper.

As shown in Fig. 2(a), the mean-field equation (3) explains the simulation results on the DRN of two CGs for any *r* very well. When 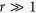, 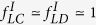 and 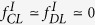 for 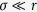. Using 
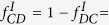
 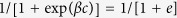
for *β* = 1 and *c* = 1 and the initial condition 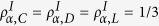, we get 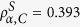 and 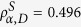 and 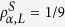 from equation (3), which explain the data for *r* < 20 in Fig. 2(a). When *r* < 20, 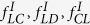 and 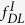 show nontrivial behavior. The equation (3) also predicts that 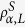 gets larger and 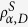 as well as 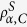 gets smaller as *r* decreases for *r* < 20.

Next, we study how the steady-state on the DRN with general 〈*k*_*intra*_〉_*α*_’s for *p* = 1 varies from the mean-field result. In Fig. 2(b,c), the simulation results on symmetric DRNs are shown. The result for large *r* (or *r* = 30) in Fig. 2(b) deviates from the mean-field result. The deviation becomes much more enhanced as 〈*k*_*intra*_〉_*α*_ gets smaller. Furthermore, 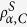 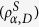 also gets larger (smaller) compared to the mean-field result. 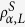 is nearly the same as the mean-field expectation for the very small 〈*k*_*intra*_〉_*α*_. The result for small *r* (or *r* = 2) in Fig. 2(c) is nearly the same as the mean-field result for small 〈*k*_*intra*_〉_*α*_. For small 〈*k*_*intra*_〉_*α*_, 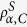 is slightly larger than the mean-field result and both 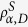 and 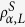 are slightly smaller than the mean-field results.

**Figure 3:**
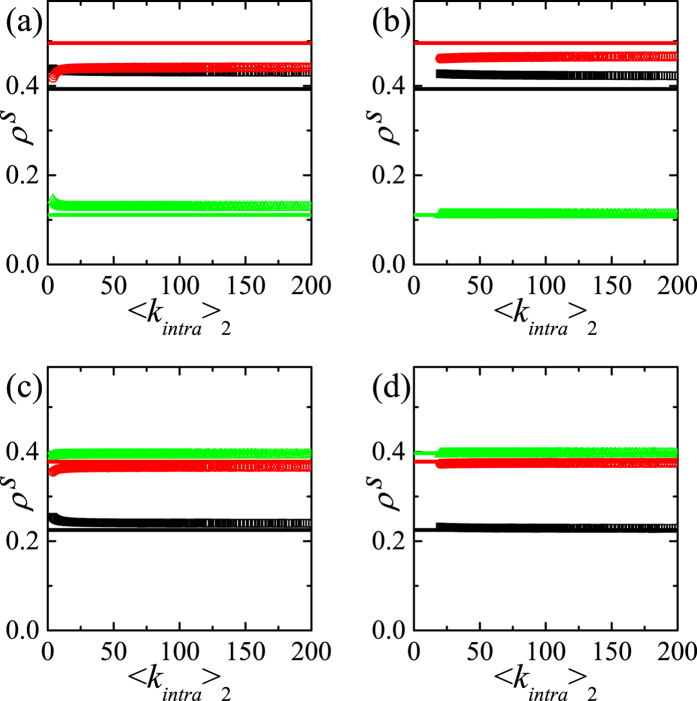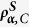, 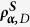 and 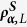 on the asymmetric DRNs with *N* = 16000 for *p* = 1. (**a**) Plots of 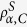, 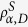 and 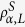 against 〈*k*_*intra*_〉_2_ and mean-field results for *r* = 30.0 and 〈*k*_*intra*_〉_1_ = 4. (**b**) Plots of 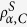, 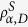 and 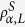 against 〈*k*_*intra*_〉_2_ and mean-field result for *r* = 30.0 and 〈*k*_*intra*_〉_1_ = 20. (**c**) Plots of 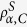, 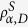 and 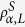 against 〈*k*_*intra*_〉_2_ and mean-field result for *r* = 2.0 and 〈*k*_*intra*_〉_1_ = 4. (**d**) Plots of 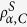, 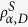 and 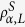 against 〈*k*_*intra*_〉_2_ and mean-field result for *r* = 2.0 and 〈*k*_*intra*_〉_1_ = 20.

In Fig. 3, the simulation results for *p* = 1 on asymmetric DRNs are shown. When *p* = 1 the interlinked pairs of the same strategies make dead pairs as in the symmetric DRN, the 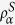
’s on both layers are the same when *N*_1_ = *N*_2_. As shown in Fig. 3, for a given 〈*k*_*intra*_〉_1_, 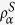
’s are nearly constant regardless of 〈*k*_*intra*_〉_2_ except for 〈*k*_*intra*_〉_2_ ≲ 〈*k*_*intra*_〉_1_. Thus, the steady-state 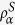
’s on the asymmetric DRNs depends mainly on 〈*k*_*intra*_〉_1_ and independent of 〈*k*_*intra*_〉_2_ except for the symmetric range 〈*k*_*intra*_〉_2_ ∼ 〈*k*_*intra*_〉_1_. For large *r (r* = 30) both 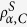 and 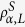 on the asymmetric DRN with the small 〈*k*_*intra*_〉_1_ (Fig. 3(a)) is larger than that on the DRN with the large 〈*k*_*intra*_〉_1_ (Fig. 3(b)), while 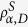 for the small 〈*k*_*intra*_〉_1_ is smaller. For small *r (r* = 2) as shown in Fig. 3(c,d), the dependences of 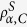 and 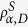 on the asymmetric DRN on 〈*k*_*intra*_〉_1_ are the same as those for large *r*. In contrast, 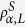 on the asymmetric DRN is nearly constant of the mean-field values regardless of 〈*k*_*intra*_〉_1_. When *r* gets larger in the interval *r* ≳ 20, 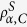 (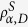) on the asymmetric DRN becomes slightly larger (smaller) than that for mean-field expectation, but 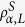 hardly varies.

Now we want to explain the theoretical origins of the results for *p* = 1 in Figs 2 and 3. As explained when deriving the mean-field result, we use the fact that the final absorbing state for *p* = 1 appears very rapidly. Thus, the initial configuration is very important to decide the final absorbing state. Topologically localized cluster of a certain strategy on CG is impossible to form if there exist Cs, Ds and Ls simultaneously. In contrast, on the layer with relatively small 〈*k*_*intra*_〉_*α*_, it is quite easy to form localized clusters of the same strategies due to fluctuation of the distribution of Cs, Ds and Ls. Such localized clusters reinforces the network reciprocity^16^,^17^,^18^,^19^,^20^,^21^,^22^,^23^,^25^. As 〈*k*_*intra*_〉_*α*_ decreases, it becomes much easier to form localized clusters. If a node *i* of a C-cluster on one layer is interlinked to a node *j* of a D-cluster on the opposite layer, then the node *j* is changed into a C-node when *P*_*i*_ > *P*_*j*_ or *r* > (*n*_*i*,*C*_ + 1)(*n*_*j*,*D*_ + 1)/[*n*_*i*,*C*_(*n*_*j*,*D*_ + 1) − (*n*_*i*,*C*_ + 1)] for *p* = 1. Since in RN *n*_*i*,*C*_ and *n*_*j*,*D*_ is roughly comparable with 〈*k*_*intra*_〉_*α*_, small 〈*k*_*intra*_〉_*α*_ enhances the fluctuation. Thus, large *r* and small 〈*k*_*intra*_〉_*α*_ make more D-nodes in D-cluster be changed into C-nodes compared to the mean-field expectation. This enhances the network reciprocity as shown in Fig. 2(b,c). If a node of a C-cluster on one layer is interlinked to a node of an L-cluster on the opposite layer, the C-node wins over the L-node for large *r*, but the L-node wins over the C-node for small *r*. Therefore, the fluctuation effect makes more C and suppresses D compared to the mean-field result and this effect becomes more enhanced as 〈*k*_*intra*_〉_*α*_ decreases. In addition, it enhances L for small *r* and suppresses L for large *r*. Thus, on the symmetric DRN, 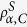 increases and 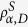 decreases as 〈*k*_*intra*_〉_*α*_ for large *r* as in Fig. 2(b). For small *r*, D-node is relatively hard to change its strategy into C. As a result the fluctuation effect is suppressed a little bit compared with large *r* and appears only on the DRN with small 〈*k*_*intra*_〉_*α*_ as in Fig. 2(c). On the asymmetric DRN, the C-cluster is more easily formed on the layer with smaller 〈*k*_*intra*_〉_*α*_ (or the layer 1), which makes the D-nodes in the opposite layer change their strategy into C. Thus the steady-state is decided by the layer 1. This effect becomes more enhanced as 〈*k*_*intra*_〉_1_ decreases. This fluctuation effect on the asymmetric DRN explains the behaviors of 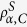 and 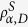 in Fig. 3. The fluctuation effect on 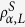 is relatively weaker as in Fig. 3.

We now explain the results for the steady-state on the asymmetric DRN for 0 < *p* < 1, which show novel network reciprocity. On the asymmetric DRN with 〈*k*_*intra*_〉_1_ and 〈*k*_*intra*_〉_2_(>〈*k*_*intra*_〉_1_), the steady-state should mainly depend on 〈*k*_*intra*_〉_1_ as expected from the result for *p* = 1. To see this effect on the asymmetric DRN we first set 〈*k*_*intra*_〉_1_ = 10, which is small enough that the steady-state on the layer 1 is the C-state when *p* = 0 (i.e., the state I and IV). If 〈*k*_*intra*_〉_2_ is also small enough, the state is the trivial C-state on both layers. If *r*_0_ < 1 and 〈*k*_*intra*_〉_2_ is in the moderately large, then the D-state (the state II) appears on the layer 2 for *p* = 0.

**Figure 4:**
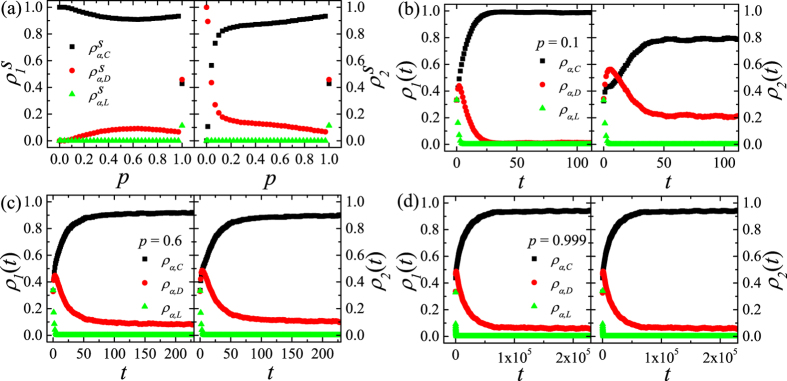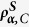, 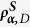 and 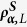 on the asymmetric DRN with 〈*k*_*intra*_〉_1_ = 10 and 〈*k*_*intra*_〉_2_ = 40 *r*_0_ = 0.3 and *N* = 16000 are used. (**a**) Plots of 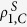, 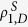 and 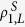 against *p* (Left) and the same plots of 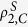, 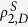 and 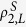 (Right) on the DRN with 〈*k*_*intra*_〉_1_ = 10 and 〈*k*_*intra*_〉_2_ = 40 for *r*_0_ = 0.3. (**b**) Time dependences of *ρ*_*α*,*C*_(*t*), *ρ*_*α*,*D*_(*t*) and *ρ*_*α*,*L*_(*t*) on *t* for *p* = 0.1. (**c**) The same plots as (**b**) for *p* = 0.6. (**d**) The same plots as (**b**) for *p* = 0.999. *t* is the Monte-Carlo time.

We first carry out the simulations on the asymmetric DRN with 〈*k*_*intra*_〉_2_ = 40 which is moderately large intradegree for *r*_0_ = 0.3(<1). As shown in Fig. 4(a), 
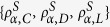
 for *p* = 0 or *p* = 1 reproduce the previously explained corresponding results very well. As *p* increases, 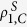 first decreases slowly from 
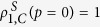
 to 
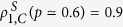
 and increases slowly to 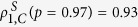. Furthermore, 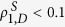 and 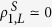 for 0 < *p* < 1. In contrast, 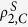 rapidly increases as *p* increases from 0 and reaches 0.8 for *p* = 0.1 and increases relatively slowly until 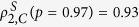. The C-dominant state on the layer 1 exists for any *p*(<1) as expected from the C-state(state I) for *p* = 0. The steady-state on the layer 2 for *p* > 0 is rather surprising, because 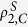 becomes very large for even very small *p*. This indicates that the inter-layer coupling drastically enhances the network reciprocity. To see the origin of this result, we study *ρ*_*α*,*C*_(*t*), *ρ*_*α*,*D*_(*t*), *ρ*_*α*,*L*_(*t*). For small *p*(=0.1), *ρ*_1,*C*_(*t*) (*ρ*_1,*D*_(*t*)) increases (decreases) rapidly at early time *t* as shown in Fig. 4(b). Due to the weak inter-layer coupling, *ρ*_1,*C*_(*t*) increases to 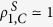 when 20 ≲ *t* as the case *p* = 0. Since the layer 2 would be in D-state when *p* = 0 as shown in Fig. 4(a), *ρ*_2,*D*_(*t*) increases and is slightly larger than *ρ*_2,*C*_(*t*) at small *t*. Through the inter-layer coupling, D’s on layer 2 can easily change their strategy into C due to the larger payoff of C on the layer 1 when *ρ*_1,*C*_(*t*) becomes large enough. As a result *ρ*_2,*C*_(*t*) increases to 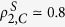 when 20 ≲ *t* ≲ 30 both *ρ*_2,*C*_(*t*) and *ρ*_2,*D*_(*t*) follow *ρ*_1,*C*_(*t*) and *ρ*_1,*D*_(*t*). Thus, for small *p*, the C-dominance on the layer 1, which is fully developed due to weak inter-layer coupling, induces the C-dominance on the layer 2 at later time. For moderate *p*(=0.6), the dependences of *ρ*_1,*C*_(*t*) and *ρ*_1,*D*_(*t*) on *t* are much more similar to those of *ρ*_2,*C*_(*t*) and *ρ*_2,*D*_(*t*) as shown in Fig. 4(c). For moderate *p*, the effects from the inter-layer coupling are nearly equal to the effect of the intra-layer interactions. Due to the increased inter-layer coupling, *ρ*_2,*C*_(*t*) increases nearly synchronously with *ρ*_1,*C*_(*t*). Such rapid increase of *ρ*_2,*C*_(*t*) makes remnant D’s on the layer 2. Those remnant D’s on the layer 2 get relatively high payoff from the intra-layer interactions with relatively dense C’s induced by inter-layer coupling. Then, through the inter-layer coupling, 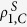 slightly decreases by remnant D’s on the layer 2 for 0.2 ≲ *p* ≲ 0.6. For large *p*(>0.6), 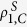 increases as *p* increases. In this case, *ρ*_*α*_(*t*)’s first reach 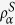 for *p* = 1 very rapidly as shown in Fig. 4(d). Then, through the intra-layer interactions, *ρ*_1,*C*_(*t*) increases very slowly from 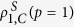, so that 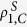 increases as *p* increases for *p* > 0.6. In contrast to the subtle dependence of 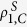 on *p*, 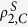 monotonically increases as *p* increases and 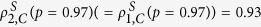. The behavior of 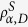 is easily understood from the behavior of 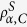 in Fig. 4(a–d). 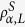 is nearly equal to 0, which can be understood from the result *p* = 0. These mechanisms explain the dependence of steady-state on *p* in Fig. 4(a) rather well.

**Figure 5:**
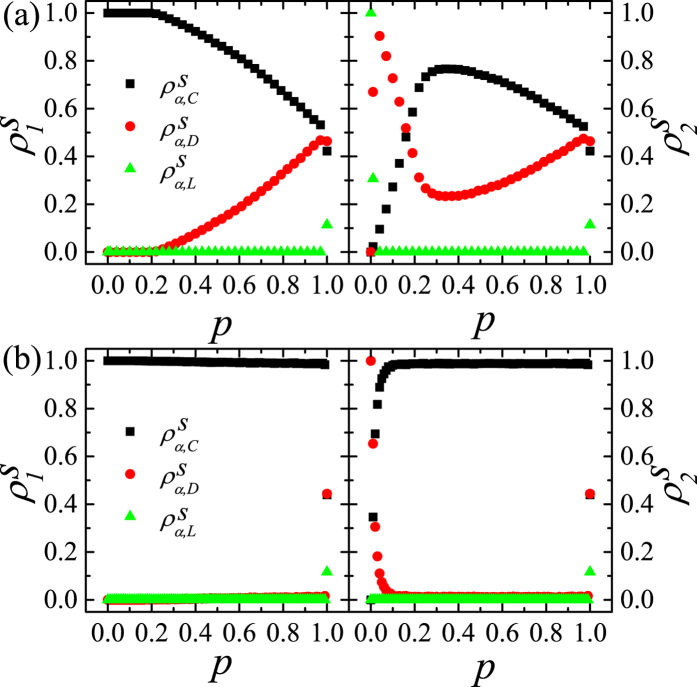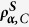, 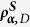 and 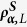 on the asymmetric DRN. *N* = 16000 are used. (**a**) Plots of 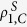, 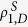 and 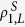 against *p* (Left) and the same plots of 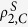, 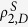 and 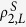 (Right) on the DRN with 〈*k*_*intra*_〉_1_ = 10 and 〈*k*_*intra*_〉_2_ = 500 for *r*_0_ = 0.3. (**b**) The same plots as (**a**) on the asymmetric DRN against *p* with 〈*k*_*intra*_〉_1_ = 100 and with 〈*k*_*intra*_〉_2_ = 2000 for *r*_0_ = 10.0.

Next, we study the inter-layer coupling effects on the asymmetric DRN with 〈*k*_*intra*_〉_1_ = 10 and 〈*k*_*intra*_〉_2_ = 500 for *r*_0_ = 0.3(<1). For *p* = 0, the steady-state on the layer 1 is the C-state (the state I) and the steady-state on the layer 2 is the L-state (the state III) as shown in Fig. 5(a). As *p* increase, 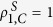 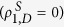 sustains for *p* < 0.2 and 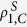 (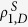) simply decreases (increases) to 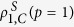 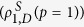. 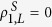 except for *p* = 1. In contrast, 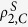 increases very rapidly for small *p* and reaches the maximum at *p* = 0.34, 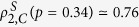. Then, 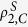 simply decreases for *p* > 0.34 to 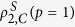. 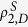 shows rather complex behavior. For very small *p*(<0.05), 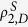 rapidly increases to the maximum, 
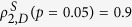
, and decreases for 0.05 < *p* < 0.34 to the minimum, 
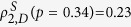
. Then, 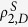 monotonically increases for *p* > 0.34 to 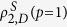. 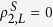 for 0 < *p* < 1. When *p* ≲ 0.34, *ρ*_*α*_(*t*)’s show nearly the same behavior as those in Fig. 4(b). For larger 〈*k*_*intra*_〉_2_ it is more difficult to make C’s through intra-layer interaction and more inter-layer coupling or large *p* is needed to increase 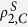. The time-dependences as in Fig. 4(b) do not occur for the larger *p*(>0.34) on the DRN with 〈*k*_*intra*_〉_1_ = 10 and 〈*k*_*intra*_〉_2_ = 500. As *p* increases further (*p* > 0.34), *ρ*_*α*_(*t*)’s behave nearly the same as in Fig. 4(c), which decreases (increases) 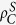
’s (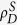
’s) on both layers. Due to large 〈*k*_*intra*_〉_2_(=500), *ρ*_*α*_(*t*)’s as in Fig. 4(d) do not happen. Instead, 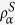
’s for larger *p* approach to 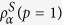
’s smoothly. These mechanisms explain the dependence of steady-state on *p* in Fig. 5(a) rather well.

We also study the SEPGG for *r*_0_ = 10.0(>1) on the asymmetric DRN with 〈*k*_*intra*_〉_1_ = 100 and 〈*k*_*intra*_〉_2_ = 2000. As shown in Fig. 5(b), the steady-state for *p* = 0 on the layer 1 is the C-state (state IV) and that on the layer 2 is the D-state (state V). For *p* > 0, 
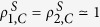
 except for very small *p*. Thus, on this DRN, *ρ*_*α*_(*t*)’s show nearly the same behavior as those in Fig. 4(b) for *p* ≠ 0 due to the very large *r*. Furthermore, the payoff of D on layer 2 is less than that of C’s in layer 1 in general. Thus, the D’s in the layer 2 easily change into C’s through the inter-layer coupling as *p* increases, which makes 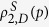 approaches to zero for *p* > 0.1.

**Figure 6:**
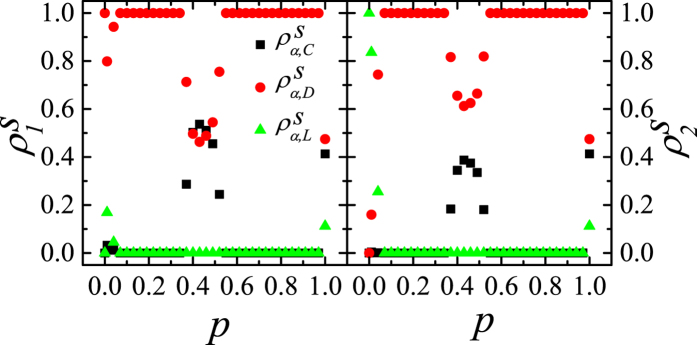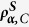, 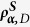 and 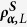 on the asymmetric DRN with 〈*k*_*intra*_〉_1_ = 40 and 〈*k*_*intra*_〉_2_ = 500. *r*_0_ = 0.3 and *N* = 16000 are used. Plots of 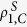, 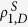 and 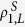 against *p* (Left) and the same plots of 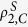, 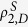 and 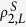 (Right) on the DRN with 〈*k*_*intra*_〉_1_ = 40 and 〈*k*_*intra*_〉_2_ = 500.

In Fig. 6 we display the behavior of 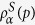
’s for *r*_0_ = 0.3(<1) on the asymmetric DRN with 〈*k*_*intra*_〉_1_ = 40 and 〈*k*_*intra*_〉_2_ = 500. As shown in Fig. 6, the steady-state for *p* = 0 on the layer 1 is the D-state (state II) and that on the layer 2 is the L-state (state III). Except for 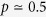, 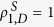 and 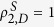. But for 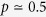, both 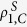 and 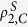 show anomalous behavior. 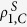 and 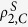 become maxima at 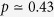 as 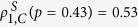 and 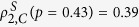. For smaller *p*(<0.4) and larger *p*(>0.6), the intra-layer interactions make 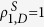 and the inter-layer couplings make 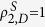. In contrast, the delicate anomalous behavior around 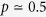 is confirmed to be originated from the balance between the inter-layer processes and the intra-layer processes, because for 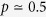, the intra-layer processes happen at nearly the same rate as the inter-layer processes. We confirm the following cyclic process I)-II)-III)-IV)-I) by simulations for 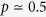. I) The intra-layer interactions on the layer 1 make C-dense regions. II) This C-dense regions induce C’s on the layer 2 through the inter-layer coupling. III) The C’s on the layer 2 make D’s by the intra-layer interactions. IV) The D’s on the layer 2 shrink the C-dense regions through the inter-layer coupling. This cyclic process makes non-zero 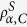 and 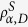 around 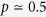. This anomalous effect also makes to appear the network reciprocity that 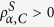 from the inter-layer coupling, without which there cannot exist any C on both layers.

We also study the model for 0 < *p* < 1 on the symmetric DRN. We check for various *p* and *r* and find that the steady-state densities on the symmetric DRN show exactly the same behavior as those on the single random network. We found that the steady-state on one layer inevitably is the same as that on the other layer. From the comparison of 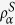
’s for various *p* and *r* to confirm that the steady-state on the symmetric DRN for 0 < *p* < 1 is exactly the same as on the corresponding single network, and find that *p* only makes the time-delay (see Supplementary Information).

### Results on the DRN with 〈*k*_*intra*_〉 < 1 or *N*_1_ ≠ *N*_2_

**Figure 7:**
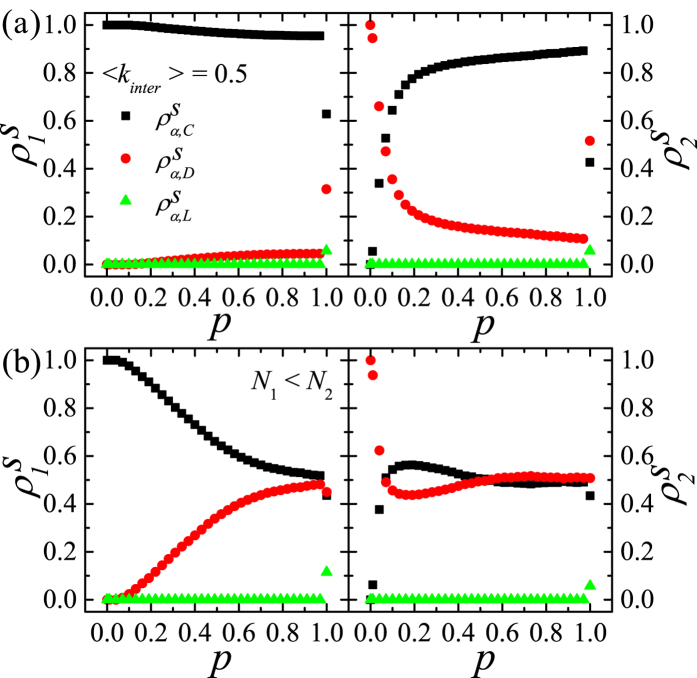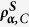, 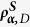 and 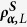 on the asymmetric DRN with 〈*k*_*intra*_〉 < 1 or *N*_1_ ≠ *N*_2_. *r*_0_ = 0.3 is used. (**a**) Plots of 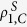, 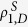 and 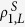 against *p* (Left) and the same plots of 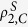, 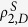 and 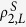 (Right) on the DRN with 〈*k*_*intra*_〉_1_ = 10 and 〈*k*_*intra*_〉_2_ = 40 for 〈*k*_*intra*_〉 = 0.5 and *N*_1_ = *N*_2_ = *N* = 16000. (**b**) The same plots as (**a**) on the asymmetric DRN against *p* with *N*_1_ = 16000, 〈*k*_*intra*_〉_1_ = 10 and with *N*_2_ = 32000, 〈*k*_*intra*_〉_2_ = 57 for *n*_*inter*_ = *N*_1_.

We also study the model on the DRN with *N*_1_ = *N*_2_ = *N* and 〈*k*_*intra*_〉 < 1. In Fig. 7(a), the results on the asymmetric DRN with 〈*k*_*intra*_〉_1_ = 10, 〈*k*_*intra*_〉_2_ = 40 and 〈*k*_*intra*_〉 = 0.5 for *r*_0_ = 0.3(<1) are shown. Comparing Fig. 7(a) to Fig. 4(a), the dependences of 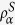
’s on *p*(<1) for 〈*k*_*intra*_〉 = 0.5 are nearly the same as those up to *p* = 0.5 for *k*_*inter*_ = 1. In contrast 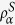
’s at *p* = 1 for 〈*k*_*intra*_〉 = 0.5 show the nontrivial behaviors, because *N*〈*k*_*intra*_〉 = 0.5*N* interlinked pairs of nodes become dead pairs in the steady-state. On the DRN with 〈*k*_*intra*_〉 < 1 the dependences of 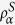
’s on *p*(<1) are generally confirmed to be nearly the same as those up to *p* = 〈*k*_*intra*_〉 except at *p* = 1.

To know the effects of the difference between two layer sizes, we study the model on the DRN with *N*_1_ ≠ *N*_2_. In Fig. 7(b), the results on the DRN with *N*_1_ = 16000, 〈*k*_*intra*_〉_1_ = 10 and *N*_2_ = 32000, 〈*k*_*intra*_〉_2_ = 57 for *r*_0_ = 0.3(<1) are shown. At *p* = 0, the steady-state of the layer 1 is the C-state (State I) and the steady-state of the layer 2 is the D-state (State II). As *p* increase, 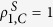 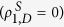 sustains for *p* < 0.05 and 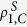 (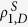) simply decreases (increases) to 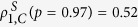 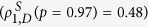. 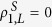 except for *p* = 1. In contrast, 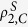 increases very rapidly for small *p*( ≲ 0.1) and reaches the maximum at *p* = 0.19, 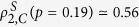. Then, 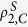 decreases slowly for *p* > 0.19 to 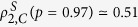. 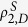 decreases very rapidly for small *p*( ≲ 0.1) and reaches the minimum at *p* = 0.19, 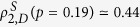. Then, 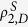 increases slowly for *p* > 0.19 to 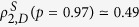. Because *N*_1_ < *N*_2_, the intra-layer interactions on the layer 2 are stronger than those for *N*_1_ = *N*_2_. The difference between the results in Fig. 7(b) and those in Fig. 5(a) are originated from the enhanced intra-layer interactions on the layer 2. Due to the enhanced intra-layer interactions in layer 2, the there are more D’s than to the case of *N*_1_ = *N*_2_ and 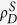
’s (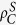
’s) on both layer increase (decrease) compared to those in Fig. 5(a). We also study the model on the DRN with *N*_1_ = 16000, 〈*k*_*intra*_〉_1_ = 40 and *N*_2_ = 32000, 〈*k*_*intra*_〉_2_ = 14 and find nearly identical results to those in Fig. 5(b). Because *N*_1_ < *N*_2_, the enhanced intra-layer interactions on the layer 2 induce the strong network reciprocity. As a result the C-state appears on both layers. We generally confirm that the intra-layer interactions on the layer with the larger size affect the steady-state considerably.

## Discussion

In summary, we study the SEPGG on DRN. When two CG’s of the same size interact through the inter-coupling with *k*_*inter*_ = 1 and *p* = 1, the steady-state density, 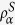 of each strategy on each layer *α* can be exactly described by the mean-field theory. If the 〈*k*_*intra*_〉_*α*_ decreases then 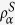
’s on each layer slightly deviates from the mean-field expectation. Such deviation is relatively small when the multiplication factor *r* is small. While if 〈*k*_*intra*_〉_1_ < 〈*k*_*intra*_〉_2_ (asymmetric DRN), then 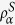
’s are determined by 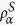
’s on the layer 1 and the network reciprocity can be reinforced through the inter-layer coupling for 0 < *p* < 1. For 0 < *p* < 1 on the symmetric DRN, 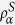
’s show exactly the same behavior with those on the corresponding single network, and *p* only makes the time-delay (see Supplementary Information). The schematic diagrams of the non-vanishing 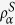
’s are also provided in Supplementary Information. Furthermore, we also investigate the behavior of 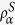
’s on the DRN with 〈*k*_*intra*_〉 < 1 or *N*_1_ ≠ *N*_2_. On the DRN with 〈*k*_*intra*_〉 < 1, we find that 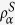
’s are nearly the same as those for 〈*k*_*intra*_〉 = 1 with *p* = 〈*k*_*intra*_〉. Finally, if *N*_1_ < *N*_2_ then the steady-state density is determined by the state of layer 2, thus the network reciprocity of the entire network can be enhanced when 〈*k*_*intra*_〉_2_ is small enough.

Furthermore, since each individual in real world interacts to each other through several different channels of interactions, such interaction topology sometimes can be well described by the multiplex networks in which all the layers have the same set of nodes in general. The DRN with *N*_1_ = *N*_2_ and *k*_*inter*_ = 1 could be related to a kind of multiplex networks. In addition, DRN with *N*_1_ ≠ *N*_2_ or *k*_*inter*_ < 1 corresponds to the interconnected networks in which each layer has different set of nodes. Therefore, our SEPGG on DRN model would provide more general framework to study the emergence of cooperation in more realistic systems.

Finally, we want to make some remarks on important open questions. Although we do not assume any detailed topological properties, many studies have revealed that some topological properties of a network such as degree heterogeneity^22^,^49^,^50^,^51^,^52^, degree-degree correlation^53^, and clustering coefficient^54^ can significantly change the evolution of cooperation. The cost heterogeneity is also known to play a nontrivial role in the emergence of cooperation^22^. As *β* changes many interesting phenomena related to the phase transition has been reported^55^,^56^. Thus it would be very important to study how such topological properties, cost heterogeneity, and noise level affect the evolution of cooperation in interlinked networks.

## Additional Information

**How to cite this article**: Kim, J. *et al*. Reciprocity in spatial evolutionary public goods game on double-layered network. *Sci. Rep.* **6**, 31299; doi: 10.1038/srep31299 (2016).

## Supplementary Material

Supplementary Information
