# Supplementary material for: Reciprocity in spatial evolutionary public goods game on double-layered network
Source: Sci Rep. 2016 Aug 9;6:31299. doi: 10.1038/srep31299 (PMC4977568; doi:10.1038/srep31299)
Supplement: Supplementary Information [file srep31299-s1.pdf]

Reciprocity in spatial evolutionary public goods game on double-layered interlinked network : Supplementary Information

Jinho Kim,[1] Soon-Hyung Yook,[1,2,*] and Yup Kim[2,†]

[1] *Department of Social Network Science, Kyung Hee University, Seoul 130-701, Korea*

[2] *Department of Physics and Research Institute for Basic Sciences, Kyung Hee University, Seoul 130-701, Korea*

[*] Corresponding author:syook@khu.ac.kr

[†] Corresponding author:ykim@khu.ac.kr

I. SYMEMETRIC DRN

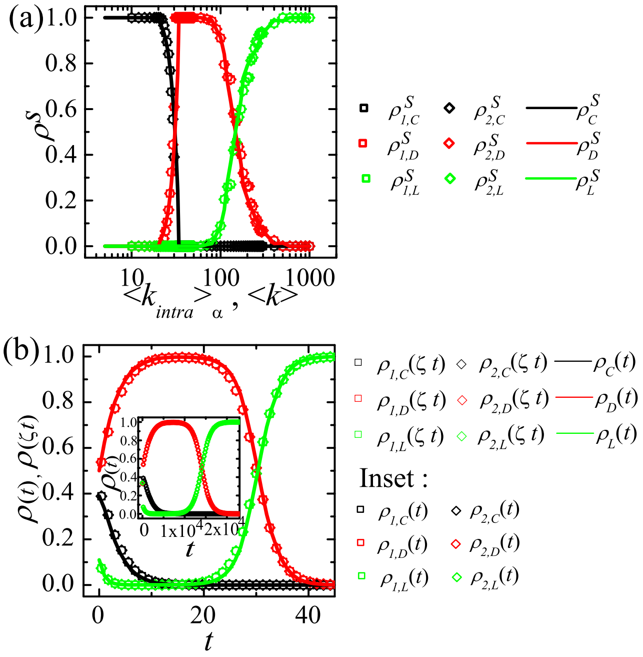

Supplementary Figure S 1. Comparison of steady-state densities on the symmetric DRN with $N = 16000$ for $p = 0.999$ to those on the single random network. $r_0 = 0.3$ is used. (a) Plots of $\rho^S_{\alpha,C}$, $\rho^S_{\alpha,D}$ and $\rho^S_{\alpha,L}$ against $\langle k_{intra}\rangle_\alpha$ (symbols) and plots of ρ^S_C, ρ^S_D and ρ^S_L against $\langle k\rangle$ of the single network(lines). (b) Plots of rescaled $\rho_{\alpha,C}(\zeta t)$, $\rho_{\alpha,D}(\zeta t)$ and $\rho_{\alpha,L}(\zeta t)$ against t for the DRN with $\langle k_{intra}\rangle_1 = \langle k_{intra}\rangle_2 = 500$ (symbols) and plots of $\rho_C(t)$, $\rho_D(t)$ and $\rho_L(t)$ against t for the single network with $\langle k\rangle = 501$. Inset of (b) Plots of $\rho_{\alpha,C}(t)$, $\rho_{\alpha,D}(t)$ and $\rho_{\alpha,L}(t)$ against t. t is the Monte-Carlo time.

We now study the model for $0 < p < 1$ on the symmetric DRN. The simulation result for $p = 0.999$ on the symmetric DRN with $N = 16000$ using the initial condition $\{\rho^I_{\alpha,C} = 1/3, \rho^I_{\alpha,D} = 1/3, \rho^I_{\alpha,L} = 1/3\}$ is shown in Fig. S1. As shown in Fig. S1(a), for $r_0 \left(\equiv rc/\sigma N^{\tanh\left(\frac{\beta c}{2}\right)}\right) = 0.3(< 1)$, the dependences of $\rho^S_{\alpha,C}$, $\rho^S_{\alpha,D}$ and $\rho^S_{\alpha,L}$ on $\langle k_{intra}\rangle_\alpha$ are exactly the same as those of ρ^S_C, ρ^S_D and ρ^S_L on $\langle k\rangle$ $(= \langle k_{intra}\rangle_\alpha + k_{i,inter}(= 1))$ of the single network. We check for various p and r and find that the steady-state densities on the symmetric DRN show exactly the same behavior as those on the single random network. To understand the origin of this result and the effect of p, we study $\rho_{\alpha,C}(t)$, $\rho_{\alpha,D}(t)$, $\rho_{\alpha,L}(t)$ for $\langle k_{intra}\rangle_\alpha = 500$. As shown in the inset of Fig. S1(b), $\rho_\alpha(t)$'s rapidly approach to ρ^S_α's for $p = 1$ at early time t, because $p = 0.999$ is so close to $p = 1$. The steady-state for $p = 0.999$

on the symmetric DRN is the L-state, which is exactly the same as the state III) for $p = 0$. To analyze the time dependence on p, we compare $\rho_\alpha(t)$'s to $\rho(t)$'s on the single network with $\langle k \rangle = 501$ using ρ^S_α's for $p = 1$ as the initial densities, i.e., $\rho(0)$'s . When one uses the time scale factor ζ that the time t_m at which $\rho_L(t_m)$ is maximal is identical to t'_m at which $\rho_{\alpha,L}(\zeta t'_m)$ is maximal, the dependences of $\rho_\alpha(\zeta t)$'s on t are nearly identical to those of $\rho(t)$'s on t as shown in Fig. S1(b). Thus, $p(< 1)$ only makes the time-delayed process to the same steady-state as on the single random network. From the comparison of ρ^S_α's for various p and r to confirm that the steady-state on the symmetric DRN for $0 < p < 1$ is exactly the same as on the corresponding single network, and find that p only makes the time-delay. It is also found that the time scale factor ζ monotonically increases as p increases. Therefore, the state for $p = 1$ on the symmetric DRN is completely unstable against p. Instead the state for $p = 0$ is stable against p. The theoretical origin of this result is as what follows. If $p < 1$, there cannot be the dead pair, because of intra-layer interactions. Since the payoff-contribution from the intralinked neighbors should be much larger than that from an interlinked neighbor, the process to the steady-state should be governed by intra-layer interaction. Since the key structures of two symmetric single layers are identical to each other, the steady-state on one layer inevitably is the same as that on the other layer. Furthermore, the state should be the same as that on the corresponding single network. Since the frequency of the update through the interlinks depends on p, p only makes the time-delay to the steady-state which depends on the intralinks.

II. SCHEMATIC DIAGRAM OF THE NON-VANISHING ρ^S_α'S

Since the ρ^S_1's are determined by ρ^S_2's when $N_1 < N_2$ (by definition we let $N_1 \le N_2$), we show the schematic diagram of ρ^S_α's only for $N_1 = N_2$ in this section. In Fig. S2 we show the schematic diagram of non-vanishing ρ^S_α's for various p when $r_0 < 1.0$. The non-vanishing strategies of each layer are separated by the slash. For example C/CD means that the C is the only non-vanishing strategy on layer 1 ($\rho^S_{1,C} = 1$) and C and D are non-vanishing strategies on layer 2 ($\rho^S_{2,C} > 0$ and $\rho^S_{2,D} > 0$). Fig. S2(a) shows the schematic diagram of ρ^S_α's for $p = 1$ at which only the interaction through the inter-layer coupling is allowed. When $\langle k_{intra} \rangle$'s in both layer become large enough ρ^S_α's are exactly the same with the mean-field results in which C, D, and L strategies coexist on both layers. As we decrease $\langle k_{intra} \rangle_\alpha$

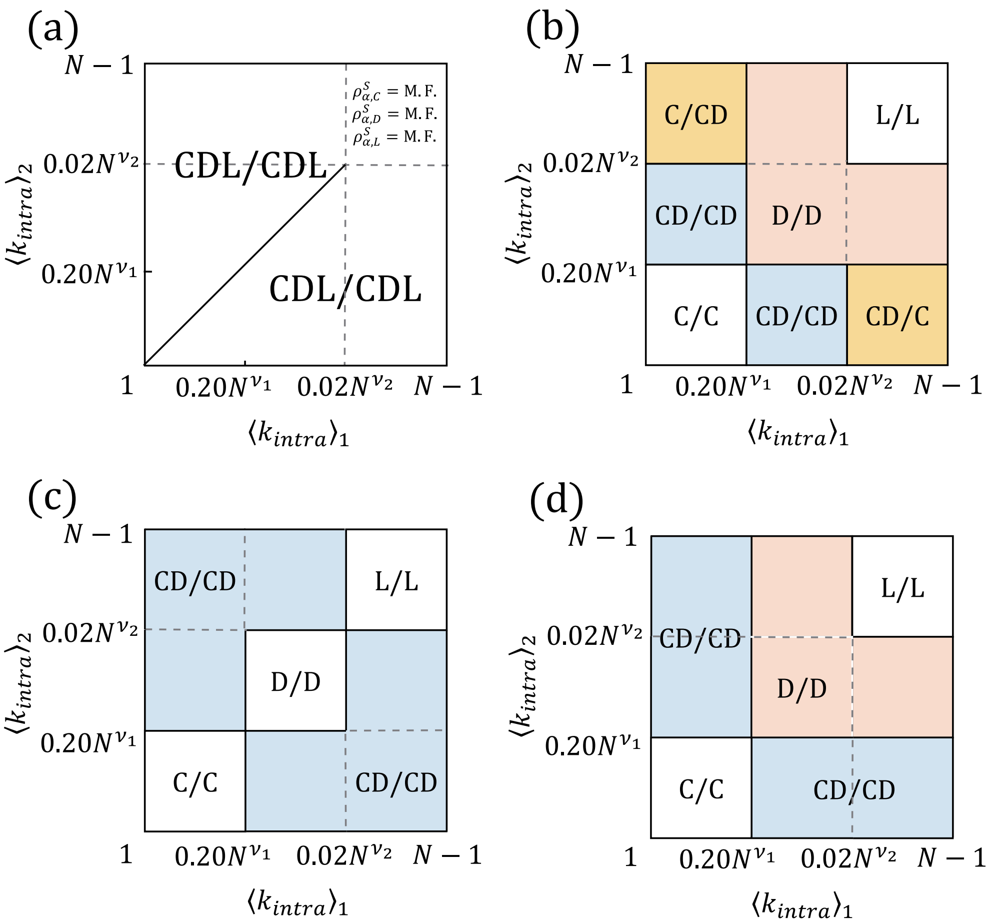

Supplementary Figure S 2. Schematic diagram of non-vanishing ρ^S_α's densities on the DRN for $r_0(\equiv rc/\sigma N^{\tanh(\frac{\beta c}{2})}) < 1.0$. **(a)** $p = 1.0$ **(b)** $0 < p \lesssim 0.3$, **(c)** $0.3 \lesssim p \lesssim 0.6$, **(d)** $0.6 \lesssim p < 1.0$. $\nu_1(\simeq 0.520)$ **and** $\nu_2(\simeq 0.898)$ **are the scaling exponents (see the main text and Ref.** [1])

to be $\langle k_{intra}\rangle_\alpha < 0.02N^{\nu_2}$ the values of ρ^S_α's deviate from the mean-field expectation but still C, D, and L strategies coexist on both layers, and ρ^S_α's are determined by the layer of smaller intradegree. In Fig. S2(b) schematic diagram of ρ^S_α's for $0 < p \lesssim 0.3$ is displayed. For $\langle k_{intra}\rangle_1 \approx \langle k_{intra}\rangle_2$ (diagonal regime in the diagram) the non-vanishing ρ^S_α's are the same with single isolated network because $N_1 = N_2$. For example, if $\langle k_{intra}\rangle_1 < 0.204N^{\nu_1}$ and $\langle k_{intra}\rangle_2 < 0.204N^{\nu_1}$, then the steady-states on both layers become the C-state, etc. As $\langle k_{intra}\rangle_1$ ($\langle k_{intra}\rangle_2$) increases while $\langle k_{intra}\rangle_2$ ($\langle k_{intra}\rangle_1$) is kept at a constant value, we find that the non-vanishing C strategy emerges on both layers which is not possible without inter-layer coupling. This indicates that the network reciprocity is significantly enhanced by the inter-layer coupling. We find the similar behavior for $0.3 \lesssim p \lesssim 0.6$ and $0.6 \lesssim p \lesssim 1.0$

as shown in Fig. S2(c) and (d), respectively.

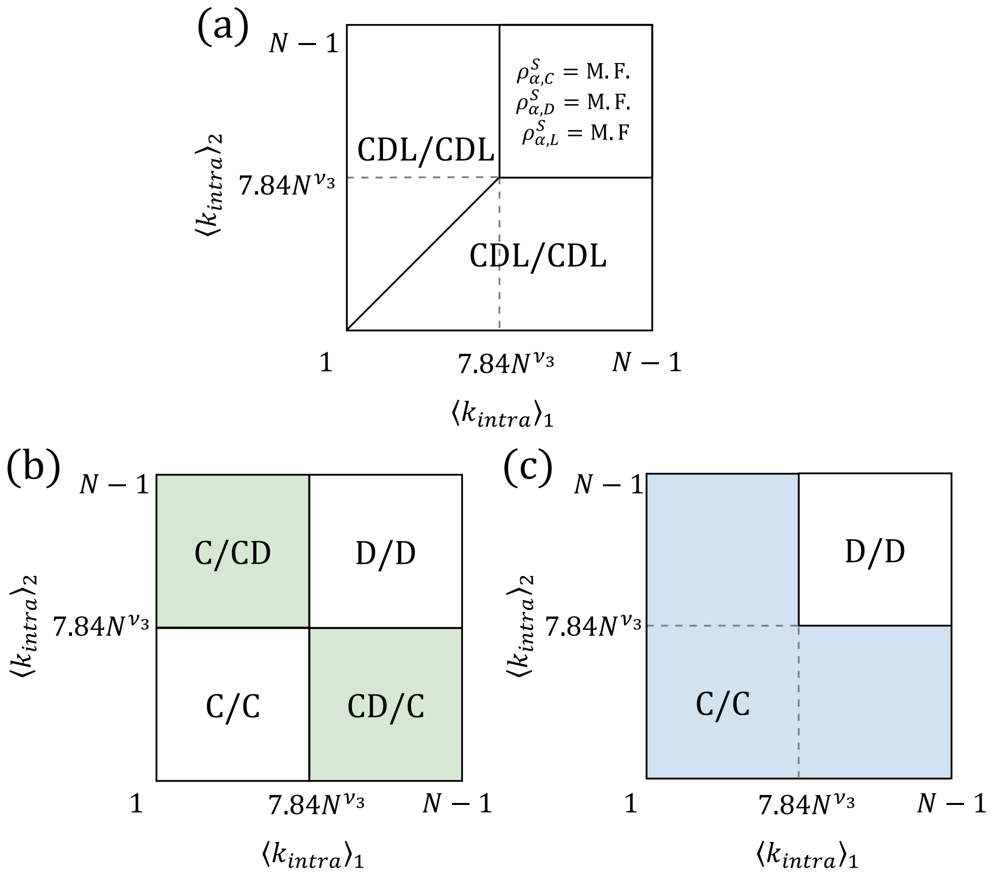

Supplementary Figure S 3. Schematic diagram of non-vanishing ρ^S_α's on the DRN for $r_0(\equiv rc/\sigma N^{\tanh(\frac{\beta c}{2})}) > 1.0$. (a) $p = 1.0$ (b) $0 < p \lesssim 0.1$ (c) $0.1 \lesssim p < 1.0$. $\nu_3 \simeq 0.51$ is the scaling exponet [1].

Fig. S3 shows non-vanishing ρ^S_α's for $r_0 > 1.0$. As shown in Fig. S3(a), if $\langle k_{intra}\rangle_1 > 7.836N^{\nu_3}$ and $\langle k_{intra}\rangle_2 > 7.836N^{\nu_3}$, then the steady-state is well described by the mean-field theory with $\rho_{\alpha,C} > 0$, $\rho_{\alpha,D} > 0$, and $\rho_{\alpha,L} > 0$. As we decrease $\langle k_{intra}\rangle_\alpha$'s to be $\langle k_{intra}\rangle_\alpha < 7.836N^{\nu_3}$, the values of ρ^S_α's deviate from the mean-field expectation, but C, D, and L strategies coexist as for the case of $r_0 < 1.0$ in Fig. S2(a). For $0 < p \lesssim 0.1$ (Fig. S3(b)) and $0.1 \lesssim p < 1$ (Fig. S3(c)), we find the similar reinforcement of network reciprocity

as for the case of $r_0 < 1.0$.

[1] Kim, J., Chae, H., Yook, S.-H. & Kim, Y. Spatial evolutionary public goods game on complete graph and dense complex networks. *Sci. Rep.* **5**, 9381 (2015).
